# 

**Published:** 1913-11-08

**Authors:** 


### BEQUESTS FOR MEDICAL STUDENTS.

Mrs. Caroline Conradine Sophia Dickinson, of Boscombe, has left £1,000 to endow a "James Bathgate Dickinson" Bed in Manchester Royal Infirmary; and £500 to endow a "James Bathgate Dickinson" Cot in the Royal Victoria and West Hants Hospital. Subject to several other bequests, she left a fund of £8,180 16s. 6d. upon trust to provide four "James Bathgate Dickinson" Scholarships for medical students at the Manchester Royal Infirmary and at Owens College, Manchester, one-fifth for a scholarship in anatomy, one-fifth for one in pathology, one-fifth for a research scholarship in surgery, and two-fifths for a research scholarship in medicine. If her estate will then admit of it, she further left £1,800 upon trust, as to one-half for a scholarship in chemistry tenable at the Stalybridge Technical School, and as to one-half upon trust for Epsom College (formerly the Royal Medical Benevolent College) for such purposes other than building as the governors may think fit.

MR. ARTHUR SAMUEL'S GIFT TO NORWICH.

Thanks to the great generosity and public spirit of the Lord Mayor of Norwich, Mr. Arthur M. Samuel, the debt on the Norfolk and Norwich Hospital, which amounted to over £5,000, has been cleared off. His recent offer to contribute £2,500 if the rest were subscribed produced an equally prompt and generous response, with the result that the hospital is now debt free.

### Appointments.

Dr. H. E. Corlin, Medical Officer of Health for Stockport, has been appointed Medical Officer of Health for Calcutta at a salary of £1,600 a year. A graduate and gold medallist of London University, and previously a scholar at St. Mary's Hospital, he has held his present post since 1908.

The following appointments have been made by the Seamen's Hospital Society:—*Dreadnought* Hospital, Greenwich: House physicians, G. Sparrow, M.R.C.S., L.R.C.P., and H. Austin Williams, M.R.C.S., L.R.C.P.; house surgeons, Douglas Ross, M.R.C.S., L.R.C.P., and J. Alban Andrews, M.B., Ch.B.; and at the Albert Dock Hospital: House surgeons, C. E. Mouat Biggs, M.R.C.S., L.R.C.P., and N. E. Seppelt, L.R.C.P. and S.

The following appointments have been made at the Hampstead and North-West London Hospital:—Ophthalmic surgeon to out-patients, Mr. T. W. Letchworth, M.B., B.C., F.R.C.S.; medical officer for *x*-ray and electrical department, Mr. W. H. Kingsbury, M.R.C.S., L.R.C.P.; house physician, Mr. O. R. L. Wilson, M.R.C.S., L.R.C.P.; house surgeon, Mr. H. O'Callaghan, M.B., Ch.B.; resident casualty officer, Mr. E. P. Scott, B.S., M.R.C.S., L.R.C.P.; and assistant casualty officer, Mr. R. K. Shaw, M.B., Ch.B.

### A HINT ON CHARITY ENTERTAINMENTS.

The Acton Cottage Hospital has always been fortunate in possessing warm supporters amongst the municipal representatives of its area of work, and the result of the recent concert held on October 15 at the Grand Hall, Acton, is further proof of this happy state of things. The concert, which was organised by Mrs. W. J. Boissonnade, wife of the Chairman of the Acton Urban District Council, Mrs. R. O. Davies, and Mrs. D. J. Thomas, wife of Dr. D. J. Thomas, medical officer of the Acton Urban District Council, was noteworthy for the fact that all the artistes, who gratuitously gave their services, were of very high standing. The financial result has now been announced, and it is stated that a cheque for £82 14s. 3d. will be handed to the Acton Cottage Hospital. Mr. Leopold de Rothschild warmly co-operated and financially assisted the concert, whilst Mr. Councillor S. M. Skinner, of the Acton Urban District Council, gave a generous donation and also supplied the piano. Mrs. W. J. Boissonnade, at the conclusion of her report upon the concert, adds: "As a hint to future promoters of charity entertainments, may I say the items for refreshments are well under 10s., alcoholic drinks standing at *nil*?"

### THE SALE OF MOUNT VERNON HOSPITAL.

The London County Council decided at its meeting on Tuesday that it is not in a position to purchase Mount Vernon Hospital, but it was agreed to call the attention of the Metropolitan Asylums Board to the proposed sale of the institution. The Public Health Committee had asked the Council to adopt this course, but in addition to express the opinion that it was desirable that the hospital should be preserved for the treatment of insured persons suffering from tuberculosis. The Council, however, declined to express any opinion on the matter, following on a speech by Mr. D. L. Courtauld, who said that the building could not be said to be suitable in every respect. The eastern portion contained three quite modern open-air wards, but the western end, built about forty years ago, was erected on the old hospital system and did not conform to the modern ideas of a sanatorium. The fact that it only had two and a half acres of ground was also against its being used as a sanatorium on a large scale. To use it as an institution for advanced cases would make it a miserable depressing place. It could cope with hospital cases perfectly well, but before long they would find that there was sufficient provision in London for that class of case. Mr. Courtauld mentioned that at present there is a vacancy for male patients in Brompton Hospital, a very unusual occurrence.

### Gifts and Bequests.

King Edward's Hospital Fund for London has been credited at the Bank of England with £500, the current year's instalment of the £2,500 voted by the Corporation of the City of London.

The result of Mme. Bernhardt's "Good Samaritan" performance at the Coliseum, at which the King and Queen were present, is announced to be that the Charing Cross Hospital and the French Hospital will benefit to the extent of at least £5,000.

Mrs. Spencer Stidolph has given a sum of £1,000 to the *Dreadnought* Hospital, Greenwich, to endow and name a bed in memory of her husband. The late Mr. E. S. Stidolph was a generous benefactor of the hospital and of every philanthropic enterprise in his neighbourhood.

A "Golden" Apple.—A giant apple was sold for 16 guineas at Covent Garden Market last week. It measured 17 inches in circumference and weighed 2 lb. 5¼ oz. It was put up for auction by Messrs. Garcia, Jacobs, Limited, and the sum obtained went in aid of Charing Cross Hospital.

